# Comparison of HIV-, HBV-, HCV- and Co-Infection Prevalence between Chinese and Burmese Intravenous Drug Users of the China-Myanmar Border Region

**DOI:** 10.1371/journal.pone.0016349

**Published:** 2011-01-21

**Authors:** Yan-Heng Zhou, Feng-Liang Liu, Zhi-Hong Yao, Lin Duo, Hong Li, Yi Sun, Yong-Tang Zheng

**Affiliations:** 1 Key Laboratory of Animal Models and Human Disease Mechanisms of Chinese Academy of Sciences and Yunnan Province, Kunming Institute of Zoology, Chinese Academy of Sciences, Kunming, China; 2 The Second People's Hospital of Yunnan Province, Kunming, China; 3 Yunnan Center for Disease Control and Prevention, Kunming, China; 4 The First People's Hospital of Yunnan Province, Kunming, China; 5 The Graduate School of the Chinese Academy of Sciences, Beijing, China; Kantonal Hospital St. Gallen, Switzerland

## Abstract

**Background:**

Co-infection with HIV and HCV and/or HBV is highly prevalent in intravenous drug users (IDUs). Because of the proximity to the “Golden Triangle”, HIV prevalence among the IDUs is very high in the China-Myanmar border region. However, there are few studies about co-infection with HIV and HCV and/or HBV, especially in the region that belongs to Myanmar.

**Methods:**

721 IDUs, including 403 Chinese and 318 Burmese, were investigated for their HIV, hepatitis B virus (HBV), and hepatitis C virus (HCV) serological status. Statistical analysis was performed to evaluate the differences of the epidemic situation between the Chinese IDUs and the Burmese IDUs.

**Results:**

Among the Chinese IDUs and the Burmese IDUs, HCV infection was the most prevalent (69.0% vs 48.1%, *P*<0.001), followed by HBV (51.6% vs 43.1%, *P*<0.05) and HIV (33.7% vs 27.0%, *P*>0.05). Besides, there were more HIV-HBV co-infected IDUs (20.1% vs 11.3%, *P*<0.005), and HIV-HCV co-infected IDUs (31.8% vs 23.9%, *P*<0.05) in China than in Myanmar, as well as HIV-HBV-HCV triple infection (19.1% vs 10.4%, *P*<0.005).

**Conclusion:**

Co-infection with HIV and HCV and/or HBV is highly prevalent among the IDUs in the China-Myanmar border region. The HIV epidemic appears to be in a downward trend, compared with previous reports. However, all infections were more prevalent among the Chinese IDUs than among the Burmese.

## Introduction

Human immunodeficiency virus (HIV), hepatitis B virus (HBV), and hepatitis C virus (HCV), the three most common chronic viral infections all over the world, share similar transmission routes including sexual, blood-blood contact, and injecting drug usage [1], [2]. Co-infection with HIV and HCV and/or HBV is very common in certain population, such as intravenous drug users (IDUs) who often share the contaminated needles/syringes for intravenous drug injection. It has been reported that the prevalence of HIV-HCV co-infection among IDUs can surpass 90% [3], [4]. Besides, the rates of HIV-HBV co-infection are reported as high as 10–20% in countries with intermediate and high HBV endemic [5]. An increasing number of studies have suggested HIV can accelerate the natural course of chronic hepatitis C or hepatitis B. Previous studies have indicated that patients with chronic viral hepatitis co-infected with HIV will experience more rapid progression and are more likely to die of liver-related diseases compared with those without HIV infection. Inversely, the effect of HCV and/or HBV on HIV infection is less clear [5]–[7]. Co-infection with HIV and hepatitis viruses has significantly increased morbidity and mortality of the HIV/AIDS patients [5], [7]. Therefore, it is critical to investigate the prevalence of co-infection with HIV and HCV and/or HBV, especially among the IDUs considered to be a high-risk population of co-infection.

China is estimated to have the world's largest population of IDUs (approximately 2.4 million) and the population of living with HIV is fairly high (6.7% to 13.4%) [8]. The HIV epidemic in China first appeared among IDUs from Dehong prefecture of the Yunnan province bordering Myanmar, which is an important transfer station for drug trafficking from the “Golden Triangle” [9], [10]. Yunnan province, located in southwest China, has become one of the most severe HIV/AIDS epidemic provinces in China, while Dehong prefecture is one of the most prevalent regions in Yunnan province [9], [11], [12]. It is estimated that there are 15,000 IDUs in Dehong prefecture, and more than half of them (54.4%) are infected with HIV [10]. The specific geographic location of Yunnan is mostly responsible for not only the high prevalence of HIV infection but also hepatitis viruses such as HCV and co-infection of them among the IDUs [8], [13]–[15]. Survey data have shown that the HCV infection is as high as 76.6% (95% CI: 66.6%–86.7%) and the HIV-HCV co-infection is more than 90% among the HIV-infected IDUs in Yunnan province [15], [16].

Myanmar, constituting the famous “Golden Triangle” with Laos and Thailand, is one of South-East Asia's countries hardest hit by the HIV epidemic, particularly in the northern region [17], [18]. Though there has been a slow decrease from 62.7% since 2000, by 2008, 37.5% IDUs were estimated to be infected with HIV, and prevalence was highest in Myitkyina (54%), located in North Myanmar, followed by Muse (43.33%) and Lashio (37.43%), both located in East Myanmar [19], [20]. The first survey undertaken by Okada *et al.* on the hepatitis C virus infection showed that 39% were HCV seropositive among patients with thalassemias and those with liver diseases in Myanmar [21]. However, there are virtually no other data regarding hepatitis virus infections among the IDUs.

The IDUs appear to have been the major group severely affected by HIV both in Myanmar and China. However, studies of co-infection with HIV and HCV and/or HBV among the IDUs in Yunnan province of China and especially Myanmar are few and even absent. Therefore, the authors have carried out a transnational study in the China-Myanmar border region to investigate and compare the epidemics of co-infection with HIV and HCV and/or HBV among IDUs in both the countries.

## Methods

### Study population

**Figure 1:**
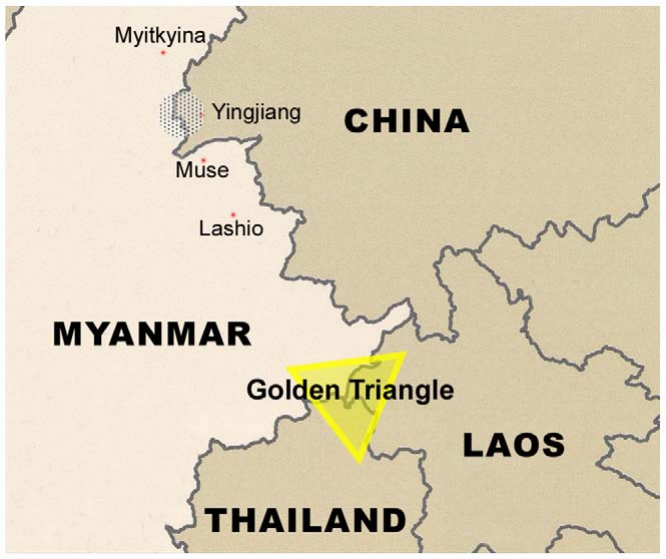
Geographic location of transnational study. 403 IDUs were recruited from Yingjiang county (shadow in the figure belonging to China), Dehong prefecture in Yunnan province of China, and 318 IDUs were from the region of northeastern Myanmar close adjacent to Dehong prefecture (shadow in the figure belonging to Myanmar).

A transnational study was carried out between March 2009 and September 2009. The study recruited 721 intravenous drug users (IDUs); 403 IDUs were from Yingjiang county, Dehong prefecture in Yunnan province, China, and 318 from northeastern Myanmar quite adjacent to Dehong prefecture (Figure 1). In China, the IDUs were recruited from community and Methadone maintenance treatment programs (MMT) with the help of the Yingjiang CDC, while all Burmese IDUs were drawn from community with the assistance of Yingjiang AIDS Prevention & Control Office, Longchuan AIDS Prevention & Control Office of Yunnan, and HU project. All volunteers provided a written informed consent and accepted a questionnaire regarding history of intravenous drug usage. After the volunteers agreed, blood was collected using vacutainer tubes. The plasma was then prepared and stored in a −70°C freezer until use according to standard procedures.

### Ethics Statement

The protocol of the study and the informed consent process were approved by the Ethics Committee of Kunming Institute of Zoology, Chinese Academy of Sciences. Written informed consent was obtained from all involved study participants.

### Virological assays

HIV status test was carried out using an enzyme-linked immunosorbent assay (ELISA, Wantai Biological Pharmacy Enterprise Co., Ltd, Beijing, China) and confirmed by another ELISA kit (Shanghai Kehua Bio-engineering Co., Ltd., Shanghai, China). The tests used to detect HBV and HCV were ELISA seropositivity for HBsAg and anti-HCV (Shanghai Kehua Bio-Engineering Co., Ltd., China).

HIV-HBV, HIV-HCV, or HBV-HCV co-infection and HIV-HCV-HBV triple infections and HIV-HCV-HBV negative were defined as positive HIV and HBV serology, positive HIV and HCV serology, positive HBV and HCV serology, positive HIV, HCV and HBV serology, and negative HIV, HCV and HBV serology results, respectively.

### Statistical analysis

Comparison of HIV, HCV, and/or HBV infection or co-infection between the Chinese and the Burmese IDUs was performed using a chi-square test or a *t* test, as appropriate. The statistical significance was fixed at *P* = 0.05. The statistical analysis was performed using the statistical software SPSS 16.0 for Windows (SPSS, Chicago, IL, USA).

## Results

### Basic information of the Chinese IDUs and the Burmese IDUs

In total, there were 403 (55.9%) Chinese IDUs and 318 (44.1%) Burmese IDUs (Table 1). On average, the Chinese IDUs (median, 32.3±8.8; range, 17 to 68 years) were 0.5 years older than the Burmese IDUs (median, 31.8±9.8; range, 17 to 78 years). Otherwise, there was no significant difference in age and the age of first drug injection between the Chinese IDUs and the Burmese IDUs. However, it was found to be significantly longer (*P*<0.001): 2 years for the Chinese IDUs' (median, 6.2±4.8; range, 0 to 24 years) injected drug usage than the Burmese (median, 4.2±4.6; range, 0 to 27 years). Few female IDUs were recruited in both China and Myanmar (1.0% VS 3.5%), and there was clear distinction of gender between the Chinese IDUs and the Burmese IDUs (*P*<0.05). In respect of ethnicity, the Han and the Dai groups were in similar proportion (41.5% VS 42.0%) and constituted the majority of the Chinese IDUs, while the Jingpo group was the biggest population (48.9%) among the Burmese IDUs, followed by the Han (29.1%) and the Dai (15.6%).

**Table 1:** The demographic character of the study participants.

Parameter	Volunteer group (no. [%])		*P*
	Chinese IDUs	Burmese IDUs	
Subjects	N = 403	N = 318	
Age (Mean±SD)	32.3±8.8^a^	31.8±9.8^b^	NS
Age of first druginjection (Mean±SD)	26.0±8.3^c^	27.4±8.6^d^	NS
Years of druginjection (Mean±SD)	6.2±4.8^e^	4.2±4.6^f^	<0.001
Gender			<0.05
Male	399 (99.0)	279 (96.5)	
Female	4 (1.0)	10 (3.5)	
Total^g^	403	289	
Ethnicity			<0.001
Han	166 (41.5)	82 (29.1)	
Dai	168 (42.0)	44 (15.6)	
Jingpo	53 (13.3)	138 (48.9)	
Other	13 (3.2)	18 (6.4)	
Total^h^	400	282	

IDUs, intravenous drug users; NS, not significant.

a data available for 400 subjects.

b data available for 289 subjects.

c data available for 391 subjects.

d data available for 240 subjects.

e data available for 385 subjects.

f data available for 242 subjects.

g Total number of subjects with known gender.

h Total number of subjects with known ethnicity.

### Prevalence of HIV, HBV, and HCV

**Figure 2:**
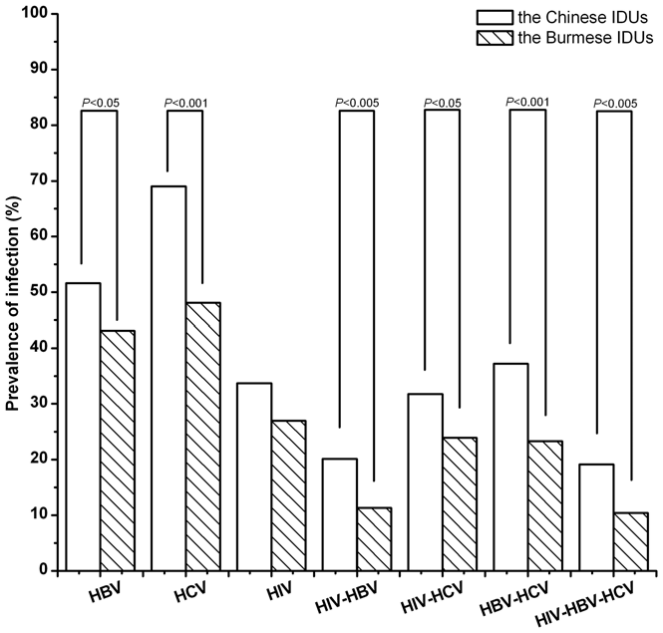
Prevalence of HBV, HCV and HCV infection or co-infection among the Chinese IDUs or the Burmese IDUs.

The infection of viruses in the Chinese and the Burmese IDUs is summarized in Table 2 and Figure 2. HCzV infection was the most prevalent in both China (69%) and Myanmar (48.1%). HIV, HCV, and HBV were more prevalent among the Chinese IDUs than among the Burmese IDUs, and the differences were statistically significant except that of the HIV epidemic. Among HIV-, HCV-, or HBV-infected IDUs, the mean age of the Chinese IDUs was higher than the Burmese IDUs, and it was statistically significant (Table 3).

**Table 2:** Prevalence of HBV, HCV, HIV infection between the Chinese IDUs and the Burmese IDUs.

Group	HBV	HCV	HIV	HIV-HBV	HIV-HCV	HBV-HCV	HIV-HBV-HCV
Chinese IDUs	208 (51.6%)^a^	278 (69.0%)^c^	136 (33.7%)	81 (20.1%)^b^	128 (31.8%)^a^	150 (37.2%)^c^	77 (19.1%)^b^
Burmese IDUs	137 (43.1%)	153 (48.1%)	86 (27.0%)	36 (11.3%)	76 (23.9%)	74 (23.3%)	33 (10.4%)

a *P*<0.05,

b *P*<0.005,

c *P*<0.001 versus Burmese IDUs.

**Table 3:** Comparison of infection between the Chinese IDUs and the Burmese male IDUs.

Parameter	HIV			HBV			HCV		
	Chinese IDUs	Burmese IDUs	*P*	Chinese IDUs	Burmese IDUs	*P*	Chinese IDUs	Burmese IDUs	*P*
Age (Mean±SD)	33.8±7.9	30.7±7.8	<0.01	34.4±9.3	32.0±9.0	<0.05	34.7±7.5	30.8±6.1	<0.05
Male Gender^a^	134/399(33.6%)	82/279(29.4%)	NS	204/399(51.1%)	123/279(44.1%)	NS	275/399(68.9%)	145/279(52.0%)	<0.001
Ethnicity									
Han^b^	48/166(28.9%)	18/82(22.0%)	NS	76/166(45.8%)	32/82(39.0%)	NS	104/166(62.7%)	31/82(37.8%)	<0.001
Dai^c^	59/168(35.1%)	8/44(18.2%)	<0.05	103/168(61.3%)	19/44(43.2%)	<0.05	119/168(70.8%)	21/44(47.7%)	<0.005
Jingpo^d^	24/53(45.3%)	41/138(29.7%)	<0.05	21/53(39.6%)	57/138(41.3%)	NS	41/53(77.4%)	73/138(52.9%)	<0.005

IDUs, intravenous drug users; NS, not significant.

a Infected male gender versus total male gender.

b Infected Han group versus total Han group.

c Infected Dai group versus total Dai group.

d Infected Jingpo group versus total Jingpo group.

Because the majority of the 721 study participants (Table 1) was male in both China (99%) and Myanmar (96.5%), the authors only compared the ratio of HIV-, HCV-, and HBV-infected or co-infected male-to-total male to avoid influence of gender on results. The results indicated that the HCV infection was more prevalent among the Chinese male IDUs than among the Burmese male IDUs (Table 3). In contrast, though more Chinese male IDUs were infected with HBV or HIV similar to HCV, they were not statistically significant (Table 3).

After comparing the epidemic among different ethnic males from China and Myanmar, the Dai or the Jingpo ethnic minority was found to be the most infected group in both the countries, especially the latter. Furthermore, all infections were significantly more prevalent among these two ethnic minorities from China than from Myanmar, except the HBV epidemic in the Jingpo ethnic group (Table 3). Besides, the rates of infection in the Han group were also remarkably high in the two countries (Table 3).

### HIV and HBV co-infection

There were 81 (20.1%) and 36 (11.3%) HIV-HBV co-infection among the Chinese IDUs and the Burmese IDUs, respectively (Table 2, Figure 2). Furthermore, the difference was statistically significant (*P*<0.005). The Chinese HIV-HBV co-infected male IDUs (34.6±7.5) were significantly older (*P*<0.01) than the Burmese (30.6±7.0) (Table 4). In the male IDUs, the HIV-HBV co-infection was more prevalent in China than in Myanmar (19.5% vs 12.9%, *P*<0.05). Among the Chinese, co-infection was most widespread in the Dai group (25%), while among the Burmese, it was in the Jingpo group (10.9%). Similar to HIV, HBV, and HCV infection situation, HIV-HBV co-infection was also significantly more prevalent among the Chinese Dai and Jingpo groups than among the Burmese (Table 4).

**Table 4:** Comparison of co-infection and triple infection between the Chinese IDUs and the Burmese male IDUs.

Parameter	HIV-HBV			HIV-HCV			HBV-HCV			HIV-HBV-HCV		
	ChineseIDUs	BurmeseIDUs	*P*	ChineseIDUs	Burmese IDUs	*P*	ChineseIDUs	Burmese IDUs	*P*	ChineseIDUs	Burmese IDUs	*P*
Age (Mean±SD)	34.6±7.5	30.6±7.0	<0.01	33.7±7.9	30.6±6.7	<0.01	34.9±9.0	31.6±7.2	<0.01	34.7±7.5	30.8±6.1	<0.05
MaleGender^a^	78/399(19.5%)	36/279(12.9%)	<0.05	127/399(31.8%)	74/279(26.5%)	NS	147/399(36.8%)	72/279(25.8%)	<0.005	74/399(18.5%)	33/279(11.8%)	<0.05
Ethnicity												
Han^b^	26/166(15.7%)	7/82(8.5%)	NS	46/166(27.7%)	15/82(18.3%)	NS	51/166(30.7%)	16/82(19.5%)	NS	25/166(15.1%)	6/82(7.3%)	NS
Dai^c^	42/168(25%)	4/44(9.1%)	<0.05	54/168(32.1%)	7/44(15.9%)	<0.05	74/168(44.0%)	9/44(20.5%)	<0.005	39/168(23.2%)	3/44(6.8%)	<0.001
Jingpo^d^	12/53(22.6%)	15/138(10.9%)	<0.05	24/53(45.3%)	39/138(28.3%)	<0.05	19/53(35.8%)	34/138(24.6%)	NS	12/53(22.6%)	14/138(10.1%)	NS

IDUs, intravenous drug users; NS, not significant.

a Infected male gender versus total male gender.

b Infected Han group versus total Han group.

c Infected Dai group versus total Dai group.

d Infected Jingpo group versus total Jingpo group.

### HIV and HCV co-infection

More Chinese IDUs (128, 31.8%) were co-infected with HIV and HCV than the Burmese (76, 23.9%) (Table 2, Figure 2). That is, HIV-HCV co-infection was more common among the Chinese IDUs than among the Burmese (*P*<0.05). The mean age of the Chinese HIV-HCV co-infected male IDUs (33.7±7.9) was also higher than the Burmese (30.6±6.7), and it was statistically significant (*P*<0.01) (Table 4). Similar to the HIV-HBV co-infection, the HIV-HCV co-infection was also more prevalent among the Chinese male IDUs than among the Burmese (31.8% vs 26.5%), but it was not statistically significant. Furthermore, the Jingpo group was seen to be the largest population co-infected with HIV and HCV both in China and Myanmar (Table 4). Though there was a lack of significant difference between the Chinese Han and the Burmese Han, the HIV-HCV co-infection was more prevalent among the Chinese ethnic minorities (Dai and Jingpo) than among the Burmese (Table 4).

### HBV and HCV co-infection

HBV-HCV co-infection (37.2%) was the most prevalent of all co-infections among the Chinese IDUs, while the ratio of the HBV-HCV co-infection among the Burmese IDUs (23.3%) was similar to the HIV-HCV co-infection (Table 2, Figure 2). Furthermore, co-infection was more prevalent among the Chinese IDUs than among the Burmese IDUs (*P*<0.001) (Table 2), and the Chinese co-infected male IDUs was older (34.9±9.0 vs 31.6±7.2, *P*<0.01) and had a higher ratio of co-infection (36.8% vs 25.8%, *P*<0.005) than the Burmese co-infected male IDUs (Table 4). There were no significant differences between the Chinese Han and the Burmese Han, or the Chinese Jingpo and the Burmese Jingpo, respectively. However, the Chinese Dai IDUs seemed to be more co-infected with HBV and HCV than the Burmese Dai IDUs (44.0% vs 20.5%, *P*<0.005). (Table 4)

### HIV, HCV, and HBV triple infection

The prevalence of triple infection was found to be 19.1% in China and 10.4% in Myanmar (Table 2, Figure 2). The most common occurrence of triple infection was similar with HIV-HBV, HIV-HCV, or HBV-HCV co-infection, i.e., it was older (34.7±7.5 vs 30.8±6.1, *P*<0.05) and had a higher ratio of males (18.5% vs 11.8%, *P*<0.05) among the Chinese IDUs than among the Burmese IDUs. The HIV-HBV-HCV triple infection was more prevalent among the Chinese Dai (23.2%) group than among the Burmese Dai (6.8%) group. (Table 4)

### HIV, HCV, and HBV negative

Among the 721 IDUs recruited in the study, there were 63 (15.6%) Chinese IDUs and 95 (29.9%) Burmese IDUs who were HIV-HCV-HBV negative, and the proportion of the Chinese IDUs was significantly lower than that of the Burmese IDUs (*P*<0.005) (Table 5). For all HIV-HCV-HBV negative IDUs, the Chinese were significantly younger than the Burmese in age (28.5±6.4 vs 33.4±11.7; *P*<0.005) and the age of first drug injection (24.3±6.3 vs 29.3±9.3; *P*<0.05). However, the times of the injection of the Chinese HIV-HCV-HBV negative IDUs and the Burmese were almost the same (3.8±3.0 vs 3.6±4.0; *P*>0.05) (Table 5).

**Table 5:** The demographic comparison between the Chinese and the Burmese HIV-HCV-HBV negative IDUs.

Parameter	Volunteer group (no. [%])		*P*
	ChineseIDUs	BurmeseIDUs	
Subjects	63 (15.6)	95 (29.9)	<0.001
Age (Mean±SD)	28.5±6.4^a^	33.4±11.7^b^	<0.005
Age of first drug injection(Mean±SD)	24.3±6.3^c^	29.3±9.3^d^	<0.05
Years of drug injection(Mean±SD)	3.8±3.0^e^	3.6±4.0^f^	NS

IDUs, intravenous drug users; NS, not significant.

a data available for 63 subjects.

b data available for 84 subjects.

c data available for 59 subjects.

d data available for 69 subjects.

e data available for 59 subjects.

f data available for 67 subjects.

## Discussion

Intravenous drug users (IDUs) appear to have been the first major group severely affected by HIV both in Myanmar and in China, particularly in Yunnan province [22], [23]. Previous studies have shown that 37.5% and 54% IDUs are infected with HIV in the two countries because of frequently sharing the needles among the users [8]. Moreover, it has been indicated that the co-infection of HIV-infected patients with hepatitis viruses especially HCV or/and HBV is very common, although the co-infection ratios vary depending on the geographic regions, risk groups, and the type of exposure involved [1], [13]–[15]. The first large outbreak of HIV in China was identified in 1989 among the IDUs in Dehong Prefecture, Yunnan province on the Myanmar border in southwest China [10]. Dehong Prefecture is proximal to one of the world's largest illicit drug production and distribution center, the “Golden Triangle”, and is an important transfer station for drug trafficking [10], [24]. Because of the geographic reasons mentioned earlier, Yunnan province is one of the most severe HIV/AIDS epidemic provinces in China, while Dehong prefecture is one of the most prevalent regions in Yunnan province [11], [12]. Therefore, a transnational study was carried out in 2009 to investigate whether there was a difference in HIV, HBV, or HCV epidemic among the IDUs from the proximal regions in China and Myanmar. 403 IDUs were recruited from Yingjiang county, Dehong prefecture and 318 from northeastern Myanmar quite adjacent to Dehong prefecture. ELISA screening showed that the infection rates among IDUs were 33.7% for HIV, 69.0% for HCV, 51.6% for HBV, 31.8% for HIV-HCV co-infection, 20.1% for HIV-HBV co-infection, 19.1% for HIV-HBV-HCV triple infection, and 15.6% for HIV-HCV-HBV negative in China; the counterparts in Myanmar were 27.0%, 48.1%, 43.1%, 23.9%, 11.3%, 10.4%, and 29.9%, respectively (Tables 2 and 5).

From the results, it is obvious that the epidemic situation of infection and co-infection of HIV, HCV and HBV was more common among the IDUs in southwest China than in northeastern Myanmar (Table 3 and 4), though the difference of the HIV epidemic between two countries is not statistically significant. There are few works about co-infection of IDUs from both two countries, especially Myanmar, but all showed HIV was very prevalent among the IDUs in both southwest China and northeast Myanmar [8], [10], [11], [17], [20]. The trends of the HIV prevalence among the two countries' IDUs indicated a decrease in the overall prevalence of HIV in recent years [11], [20]. In Myanmar, the HIV prevalence among the IDUs peaked in the early 1990s at over 70% before beginning a slow but steady decline during 2005–2006 [20]. The HIV prevalence among the Burmese IDUs in 2008 was 37.5% (range: 37.2–54%), in which Myitkyina located in north Myanmar is 54%, while Muse and Lashio, both located in the east Myanmar, are 43.33% and 37.43%, respectively [19], [20]. The present study found 27% northeastern Myanmar IDUs was infected with HIV (Table 2), which was consistent with the trend. In Yunnan province, located in southwest China, the average prevalence rate among the IDUs increased from 1992 to 2004 (2.7% in 1992, 15% in 1995, 30% in 1999, 32.4% in 2004) and decreased to 28.4% in 2007; while in 7 counties, including Yingjiang, the HIV prevalence rates among the IDUs exceeded 40% [11]. The results show 33.7% of IDUs in Yingjiang county was HIV positive (Table 2), which was lower than the 52% prevalence in 2005 [10]. It was also in accord with that in Yunnan province.

HIV, HBV, and HCV have similar routes of transmission, such as sharing of needles to inject drugs [1], [2]. Among the IDUs in Myanmar, 31% in Yangon, 22% in Myitkyina, and 19% in Lashio reported sharing of needle during the last injection in 2008, respectively [20]. Surveys in China indicated that 40% of the IDUs engage in needle-sharing behavior [12], [13]. Moreover, the present analysis showed that the Chinese IDUs injected drugs for a longer time than the Burmese, though the former were younger than the latter and earlier in first usage of drug injection. Many studies concluded that the high prevalence of HIV and HCV among IDUs was associated with the duration of injection of drug usage [25], [26]. More frequent needle sharing and longer time of drug injection may be the reason why HIV, HBV, and HCV were more prevalent in China than in Myanmar in this study.

**Figure 3:**
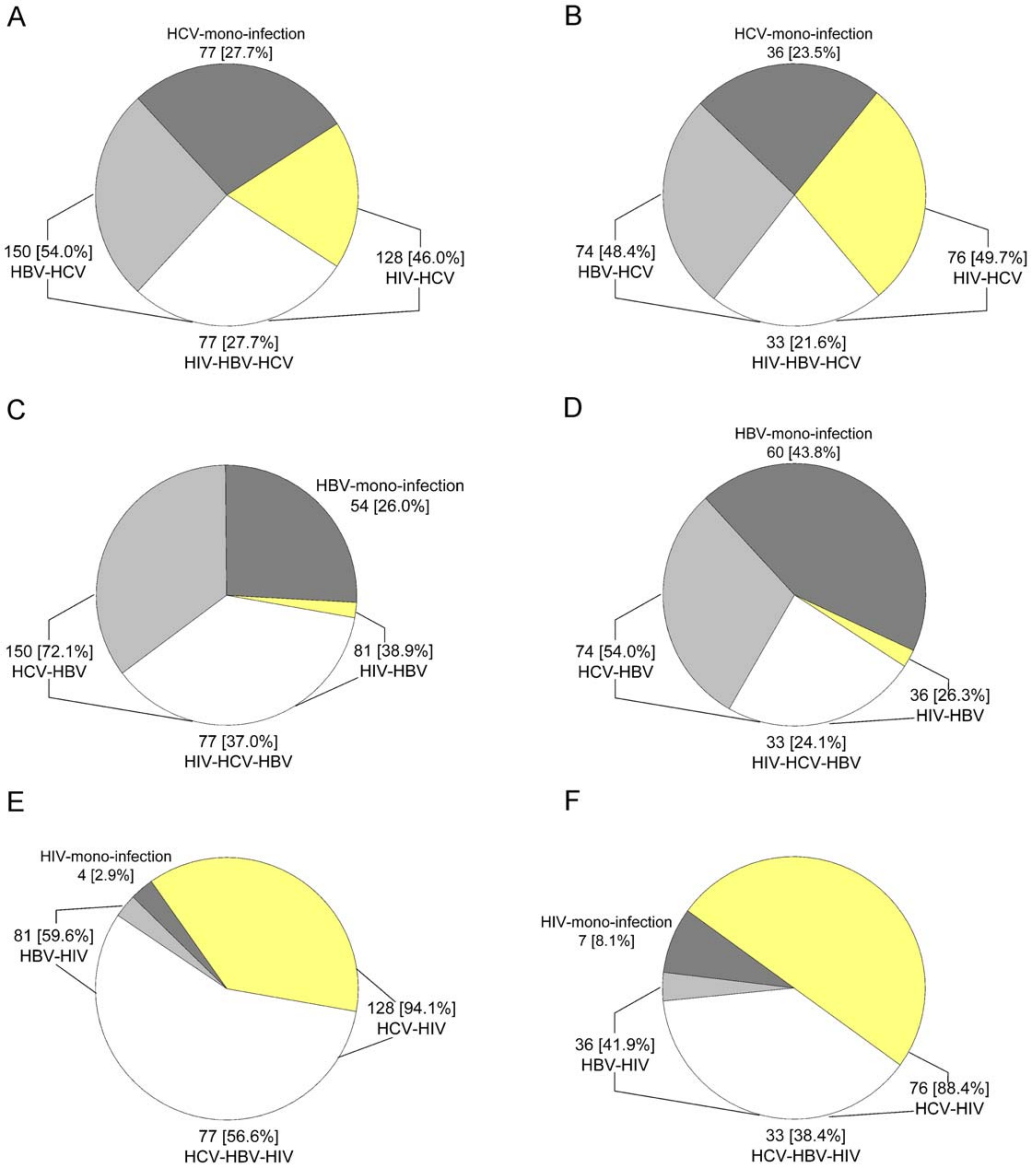
Proportion with mono-infection or co-infection among all HCV, HBV or HIV infected IDUs from China or Myanmar. proportion with HCV mono-infection, HBV-HCV co-infection, HIV-HCV co-infection and HIV-HBV-HCV triple infection among all Chinese (A) or Burmese (B) HCV infected IDUs; proportion with HBV mono-infection, HCV-HBV co-infection, HIV-HBV co-infection and HIV-HCV-HBV triple infection among all Chinese (C) or Burmese (D) HBV infected IDUs; proportion with HIV mono-infection, HCV-HIV co-infection, HBV-HIV co-infection and HCV-HBV-HIV triple infection among all Chinese (E) or Burmese (F) HIV infected IDUs.

Rates of HBV and HCV infection are higher than those for HIV in both China and Myanmar (Table 2, Figure 2), which is consistent with the previous reports [27]. HCV infection in IDUs was the highest in both the countries, compared with other infections (Table 2, Figure 2), which may be associated with the fact that IDUs acquired HCV more rapidly than HIV and HBV after starting to use drugs [28]. Evidence from needle-stick injury studies suggested the injection-related transmission probability for HCV transmission was up to 10 times greater than HIV [29]. The former can be acquired after the onset of injection drug usage through sharing infected needles, whereas HIV is infected after increasing durations of injection drug usage [30]. Studies of the impact of needle exchange program on the incidence of HIV and hepatitis infection in the USA showed that the needle exchange program participation was associated with 33% HIV infection and more than 80% HCV or HBV infection reduction [31]. In other words, the IDUs were more prone to be infected with HCV or HBV, compared to HIV, which was similar to the present study's result with mono-infection or co-infection among all HCV, HBV, or HIV infection. The proportion with HIV mono-infection among HIV-infected IDUs was significantly lower than the HCV mono-infection or the HBV mono-infection among the HCV-infected or the HBV-infected IDUs from both China and Myanmar (Figure 3). The result means that once infected with HIV, the patients had been HCV and/or HBV positive. Meantime, both China and Myanmar are HBV highly prevalent (≥8%) countries [32]. These may be the reasons why the HBV infection was also higher than HIV in this study.

The prevalence of HIV, HCV, and HBV infection and co-infection was significantly higher in ethnic minorities (Dai and Jingpo) than in the Han ethnic group (Tables 3 and 4), which is consistent with the situation of the HIV infection among Yunnan province [11]. In Yunnan province, HIV prevalence among the IDUs was higher for ethnic minorities, including Jingpo, Dai, and Yi, than for the Han ethnic group, even after controlling for different levels of education across ethnicities [11]. Though the exact reason was not known, it might be because of limited access to health care, habits, and lower public health awareness leading to prevalence in the ethnic minorities.

In conclusion, this study indicates that the prevalence of co-infection with HIV and HCV and/or HBV is very common among IDUs both in southwest China and northeast Myanmar. However, the HIV epidemic appears to be in a downward trend, compared with previous studies, though the epidemic in China is more severe than in Myanmar. This decrease is a response with harm reduction programs conducted in both two countries. In China, the first needle and syringe programs (NSP) were initiated in 1999 in Yunnan and Guangxi provinces [27]. The year 2004 was very important in China HIV/AIDS prevention and treatment. Many counseling and harm reduction programs, for example, free HIV Voluntary Counseling and Testing (VCT) and Methadone maintenance treatment programs (MMT), were launched [11], [27], [33]. A report showed that the HIV-infected ratio among the IDUs from Yunnan province peaked in 2004, and then started to decrease [11], which means that harm reduction programs were very useful in reducing HIV prevalence among IDUs. However, one study demonstrated that without such programs, the HIV prevalence among the IDUs can rise to 40% or more within 1 or 2 years after the virus is introduced in the communities [19]. This implies that the two countries need to enhance harm reduction programs continuously, to avoid increasing the HIV, HBV, and HCV prevalence among the IDUs again.

## References

[1] Saravanan S, Velu V, Kumarasamy N, Nandakumar S, Murugavel KG (2007). Coinfection of hepatitis B and hepatitis C virus in HIV-infected patients in south India.. World J Gastroenterol 2007.

[2] Koziel MJ, Peters MG (2007). Viral hepatitis in HIV infection.. N Engl J Med.

[3] Maier I, Wu GY (2002). Hepatitis C and HIV co-infection: a review.. World J Gastroenterol.

[4] Aceijas C, Rhodes T (2007). Global estimates of prevalence of HCV infection among injecting drug users.. Int J Drug Policy.

[5] Thio CL (2009). Hepatitis B and human immunodeficiency virus coinfection.. Hepatology.

[6] Sulkowski MS (2008). Viral hepatitis and HIV coinfection.. J Hepatol.

[7] Rotman Y, Liang TJ (2009). Coinfection with hepatitis C virus and human immunodeficiency virus: virological, immunological, and clinical outcomes.. J Virol.

[8] UNAIDS (2009). AIDS Epidemic Update 2009.. http://www.unaids.org/en/KnowledgeCentre/HIVData/EpiUpdate/EpiUpdArchive/2009/default.asp.

[9] Zhang Y, Lu L, Ba L, Liu L, Yang L (2006). Dominance of HIV-1 subtype CRF01_AE in sexually acquired cases leads to a new epidemic in Yunnan province of China.. PLoS Med.

[10] Jia Y, Sun J, Fan L, Song D, Tian S (2008). Estimates of HIV prevalence in a highly endemic area of China: Dehong Prefecture, Yunnan Province.. Int J Epidemiol.

[11] Jia M, Luo H, Ma Y, Wang N, Smith K (2010). The HIV epidemic in Yunnan Province, China, 1989-2007.. J Acquir Immune Defic Syndr.

[12] Wang L, Wang N, Wang L, Li D, Jia M (2009). The 2007 Estimates for People at Risk for and Living With HIV in China: Progress and Challenges.. J Acquir Immune Defic Syndr.

[13] Bao YP, Liu ZM (2009). Systematic review of HIV and HCV infection among drug users in China.. Int J STD AIDS.

[14] Wang YC, Xu SH, Li XH, Song AJ, Jia XR (2006). A study on the prevalence rates of human immunodeficiency virus, hepatitis B virus and hepatitis C virus infections in intravenous drug users.. Zhonghua Liu Xing Bing Xue Za Zhi.

[15] Zhang C, Yang R, Xia X, Qin S, Dai J (2002). High prevalence of HIV-1 and hepatitis C virus coinfection among injection drug users in the southeastern region of Yunnan, China.. J Acquir Immune Defic Syndr.

[16] Xia X, Luo J, Bai J, Yu R (2008). Epidemiology of hepatitis C virus infection among injection drug users in China: Systematic review and meta-analysis.. Public Health.

[17] Kusagawa S, Sato H, Watanabe S, Nohtomi K, Kato K (1998). Genetic and serologic characterization of HIV type 1 prevailing in Myanmar (Burma).. AIDS Res Hum Retroviruses.

[18] Williams B, Baker D, Bühler M, Petrie C (2008). Increase coverage of HIV and AIDS services in Myanmar.. Confl Health.

[19] Sharma M, Oppenheimer E, Saidel T, Loo V, Garg R (2009). A situation update on HIV epidemics among people who inject drugs and national responses in South-East Asia Region.. AIDS.

[20] United Nations Office on Drugs and Crime (UNODC) (2010). Myanmar Country Advocacy Brief Injecting Drug Use and HIV.. http://www.unodc.org/documents/eastasiaandpacific//topics/hiv-aids/UNRTF/Mya_CAB_04_Feb_10_.pdf.

[21] Okada S, Taketa K, Ishikawa T, Koji T, Swe T (2000). High prevalence of hepatitis C in patients with thalassemia and patients with liver diseases in Myanmar (Burma).. Acta Med Okayama.

[22] Liu P, Xiang K, Tang H, Zhang W, Wang X (2008). Molecular epidemiology of human immunodeficiency virus type 1 and hepatitis C virus in former blood donors in central China.. AIDS Res Hum Retroviruses.

[23] Motomura K, Kusagawa S, Kato K, Nohtomi K, Lwin HH (2000). Emergence of new forms of human immunodeficiency virus type 1 intersubtype recombinants in central Myanmar.. AIDS Res Hum Retroviruses.

[24] Chu TX, Levy JA (2005). Injection drug use and HIV/AIDS transmission in China.. Cell Res.

[25] Zamani S, Radfar R, Nematollahi P, Fadaie R, Meshkati M (2010). Prevalence of HIV/HCV/HBV infections and drug-related risk behaviours amongst IDUs recruited through peer-driven sampling in Iran.. Int J Drug Policy.

[26] Solomon SS, Srikrishnan AK, Mehta SH, Vasudevan CK, Murugavel KG (2008). High prevalence of HIV, HIV/hepatitis C virus coinfection, and risk behaviors among injection drug users in Chennai, India: a cause for concern.. J Acquir Immune Defic Syndr.

[27] Sullivan SG, Wu Z (2007). Rapid scale up of harm reduction in China.. Int J Drug Policy.

[28] Estrada AL (2002). Epidemiology of HIV/AIDS, hepatitis B, hepatitis C, and tuberculosis among minority injection drug users.. Public Health Rep.

[29] Vickerman P, Hickman M, May M, Kretzschmar M, Wiessing L (2010). Can hepatitis C virus prevalence be used as a measure of injection-related human immunodeficiency virus risk in populations of injecting drug users? An ecological analysis.. Addiction.

[30] Garten RJ, Zhang J, Lai S, Liu W, Chen J (2005). Coinfection with HIV and hepatitis C virus among injection drug users in southern China.. Clin Infect Dis.

[31] Vlahov D, Junge B (1998). The role of needle exchange programs in HIV prevention.. Public Health Rep.

[32] Liaw YF (2009). Antiviral therapy of chronic hepatitis B: opportunities and challenges in Asia.. J Hepatol.

[33] Reid G, Aitken C (2009). Advocacy for harm reduction in China: a new era dawns.. Int J Drug Policy.

